# Canonical amplifications and *CDKN2A/B* loss refine *IDH1/2*-mutant astrocytoma prognosis

**DOI:** 10.1093/neuonc/noae258

**Published:** 2024-11-25

**Authors:** Hia S Ghosh, Ruchit V Patel, Elizabeth B Claus, Luis Nicolas Gonzalez Castro, Patrick Y Wen, Keith L Ligon, David M Meredith, Wenya Linda Bi

**Affiliations:** Department of Neurosurgery, Brigham and Women’s Hospital, Boston, Massachusetts, USA; Harvard Medical School, Boston, Massachusetts, USA; Department of Neurosurgery, Brigham and Women’s Hospital, Boston, Massachusetts, USA; Department of Biostatistics, Yale School of Public Health, New Haven, Connecticut, USA; Department of Neurosurgery, Brigham and Women’s Hospital, Boston, Massachusetts, USA; Center for Neuro-Oncology, Dana-Farber Cancer Institute, Boston, Massachusetts, USA; Department of Neurology, Brigham and Women’s Hospital, Boston, Massachusetts, USA; Center for Neuro-Oncology, Dana-Farber Cancer Institute, Boston, Massachusetts, USA; Department of Pathology, Brigham and Women’s Hospital, Dana-Farber Cancer Institute, Boston, Massachusetts, USA; Department of Pathology, Brigham and Women’s Hospital, Dana-Farber Cancer Institute, Boston, Massachusetts, USA; Department of Neurosurgery, Brigham and Women’s Hospital, Boston, Massachusetts, USA

**Keywords:** *CDKN2A/B* loss, focal amplification, glioma, *IDH1/2*-mutant astrocytoma, molecular risk stratification

## Abstract

**Background:**

Molecular features have been incorporated alongside histologic criteria to improve glioma diagnostics and prognostication. *CDKN2A/B* homozygous-loss associates with worse survival in *IDH1/2*-mutant astrocytomas (*IDHmut-*astrocytomas), the presence of which denotes a grade 4 tumor independent of histologic features. However, no molecular features distinguish survival amongst histologically defined grade 2 and 3 *IDHmut-*astrocytomas.

**Methods:**

We assembled a cohort of patients ≥19 years old diagnosed with an *IDHmut-*astrocytoma between 1989 and 2020 from public datasets and several academic medical centers. Multivariate modeling and unbiased clustering were used to stratify risk.

**Results:**

We identified 998 *IDHmut*-astrocytoma patients (41.5% female; 85.6% white). Tumor grade, *CDKN2A/B* loss, and/or ≥1 focal amplification were associated with reduced survival. Grade 2/3 patients with intact *CDKN2A/B* and no focal amplifications survived the longest (OS 205.7 months). Survival for grade 2/3 cases with either *CDKN2A/B* hemizygous-loss or focal amplifications (80.4, 88.7 months respectively) did not differ significantly from grade 4 cases with intact *CDKN2A/B* and no amplifications (91.5 months, *P* = .93). Grade 4 patients with either hemizygous or homozygous loss of *CDKN2A/B* had the shortest survival (OS 31.9, 32.5 months respectively), followed by grade 4 cases with intact *CDKN2A/B* and focal gene amplifications (OS 55.9 months). Integrating *CDKN2A/B* status and amplifications alongside histopathologic grade refined overall survival prediction. Unbiased clustering revealed 9 distinct molecular profiles, with differential survival. *IDHmut*-astrocytomas with any *CDKN2A/B* loss clustered together, regardless of grade, and exhibited the poorest outcomes.

**Conclusions:**

Combining *CDKN2A/B* hemizygous-loss and focal gene amplifications reveals a group of *IDHmut*-astrocytoma patients with an intermediate prognosis, refining *IDH*mut-astrocytoma classification.

**Key Points**

1. Hemizygous *CDKN2A/B* loss predicts shorter survival in *IDH1/2*-mutant astrocytoma.
2. *CDKN2A/B* loss (homozygous or hemizygous) and focal amplifications improve risk stratification of *IDH1/2*-mutant astrocytoma patients when integrated with tumor grade.

**Importance of the Study**

While molecular classification of gliomas has enhanced diagnostics and prognostication for *IDH1/2*-mutant astrocytomas, there remains significant heterogeneity in clinical outcomes across cases with similar histopathologic grades. Certain molecular features have been proposed to help distinguish tumor grades, such as homozygous *CDKN2A/B* loss being sufficient for grade 4 designation. We sought to understand if additional molecular alterations can refine risk stratification and prognostication. Using a multi-institutional, clinically heterogenous cohort of *IDH1/2*-mutant astrocytomas, we demonstrate that *CDKN2A/B* homozygous and hemizygous loss along with focal gene amplifications negatively impact overall survival, irrespective of grade. Integrating these molecular alterations with histopathologic grade defines groups of patients with short-, intermediate-, and long-term survival. Readily accessible molecular features can help refine prognostication of overall patient survival across *IDH1/2*-mutant astrocytomas compared to the current standard, an important step toward guiding treatment strategies and clinical trial stratification.

*IDH1/2*-mutant astrocytomas exhibit significant heterogeneity in clinical behavior, with overall survival ranging from years to decades after diagnosis.^1^ Histologically similar cases often exhibit disparate clinical outcomes, even when similar treatment paradigms are employed. While *IDH1/2*-mutant astrocytomas were previously graded solely on histopathologic features, the World Health Organization (WHO) 2021 and cIMPACT-NOW update 5 have integrated molecular criteria to refine the prediction of prognosis.^1–3^ Despite these advances, it remains difficult to stratify risk between grade 2 and grade 3 *IDH1/2*-mutant astrocytomas, with weak distinction in their clinical outcomes.^4–7^ As new treatments for *IDH1/2-*mutant gliomas expand routine clinical practice, tumor classification that reflects clinical phenotype becomes critical for patient selection and assessment of therapeutic benefit.

To date, homozygous *CDKN2A/B* loss is negatively prognostic in *IDH1/2*-mutant astrocytoma patients and is sufficient for grade 4 designation.^1,3^ Recent literature suggests this association with poorer prognosis may extend to *IDH1/2*-mutant astrocytoma with hemizygous *CDKN2A/B* loss, independent of grade.^7–9^ Focal gene amplifications, a phenomenon seen in many cancer subtypes, may provide an additional marker.^10^ Amplifications in canonical prognostic genes for glioma such as *CCND2* have been linked to high histological grade and malignant tumor transformation.^11–13^ Here, we assess the prognostic impact of hemizygous *CDKN2A/B* loss as well as focal amplifications in the absence of *CDKN2A/B* loss to improve the prediction of patient survival both within and across tumor grades, while highlighting molecular patterns that may contribute to survival differences.

## Methods

### Cohort and Classification

Molecular and clinical data for adult patients ≥19 years old were gathered from three glioma datasets: (1) Dana-Farber Cancer Institute/Brigham and Women’s Hospital (DFCI); (2) Project Genomics Evidence Neoplasia Information Exchange (GENIE)^14^; and (3) The Cancer Genome Atlas (TCGA).^15^ GENIE and TCGA data were downloaded from online repositories; DFCI/BWH data were collected through chart review and institutional next-generation sequencing.^16^ Comprehensive description of data processing, molecular profiling, and germline variant filtering can be found in Supplementary Material 1. This study was approved by the Institutional Review Board of the Dana-Farber/Harvard Cancer Center.

Using the WHO 2021 and cIMPACT-NOW guidelines, *IDH1/2*-mutant astrocytomas were defined as *IDH1/2*-mutant gliomas without chromosomal 1p/19q-codeletion.^1,3^ If 1p/19q status could not be ascertained, the presence of *ATRX* or *TP53* mutations indicated it was likely an astrocytoma.^3^ Duplicate samples and those with incomplete molecular profiling were removed. For patients with multiple reported samples, the earliest profiled tumor sample was selected. We selected cases annotated as WHO grade 2, 3, or 4, excluding nonspecific grade annotations (high, low, or unknown). However, tumors with nonspecific grade annotations were considered grade 4 if they harbored *CDKN2A/B* homozygous deletion.^1,3^

*CDKN2A/B* status was categorized as homozygous loss, heterozygous loss, or intact. Focal amplification events were examined in the following canonically amplified genes reported in WHO 2021 and cIMPACT-NOW update 5: *CCND2, CDK4/6, EGFR, MDM2/4, MET,* and *PDGFRA*. Focal amplifications have a low prevalence and frequently co-occur. As a result, cases with one or more focal amplifications were treated as amplified. Other amplification events such as *MYCN* were considered but not included following a backward elimination approach using overall survival stratification as the endpoint. Focal gene amplification calls were derived from gene-specific copy number reporting from targeted next-generation sequencing in the non-TCGA cohort and through whole-exome sequencing in the TCGA cohort. Amplification events were defined as copy count ≥6 in the DFCI cohort and Memorial Sloan Kettering subset of GENIE samples.^17,18^ Copy count thresholds for amplification event annotation were not available for other GENIE cohorts and TCGA. Focal amplification events were defined as amplifications isolated to the gene of interest and/or involving closely neighboring genes. Annotated lower-level amplification events were excluded.^19,20^ All assays used to call focal amplifications as part of this study were Clinical Laboratory Improvement Amendments certified. Across both DFCI and GENIE cohorts, methodology noted pathologist review to confirm tumor cellularity at >10%.

Statistical comparison of demographic data and molecular alterations across *IDH1/2*-mutant astrocytoma stratified by grade or cohort was performed using Chi-square and proportion tests with Holm-Bonferroni correction at a *P* < .05 significance level.

### Survival Analysis

Overall survival for patients with *IDH1/2*-mutant astrocytoma was examined using Kaplan–Meier curves, with log-rank tests performed to detect a significant difference in survival at *P* < .05. Survival was measured from the date of surgery. If the date of surgery was not available, the date of diagnosis was used. The upper limit of survival was established as the time when patients were deceased or when the last follow-up was performed.

Clinical and molecular features were evaluated for prognostic value through initial univariate feature selection (*q* < 0.2 after multiple comparisons correction) followed by multivariate-adjusted Cox regression models significant at *P* < .05. Only the DFCI/GENIE datasets (non-TCGA) were used for feature selection: the TCGA dataset was excluded as TCGA overall survival was significantly shorter than that of more contemporary non-TCGA cohorts (Supplementary Material 2).^7^ Along with molecular features, Cox models were adjusted for clinical features including age, sex, race, receipt of chemotherapy, and tumor grade. As the extent of tumor resection was not available in the GENIE dataset, it was not included as a covariate. To prevent overfitting, we performed stepwise backward elimination and removed features with undefined confidence intervals. Multivariate models were internally validated through a k-fold approach (15 folds), using subsets without replacement (65% of the whole dataset) and comparison using pseudo-R^2^ (RD) model values.

### Molecular Clustering

Unsupervised uniform manifold approximation projection (UMAP) and density-based spatial clustering (DBSCAN) were used to identify clusters of *IDH1/2*-mutant astrocytoma from mutations, copy-number alterations, and structural variants in 33 genes and 10 chromosome arms which are signatures of adult-type gliomas and/or associated with prognosis. Genes included *APC, ARID1A, ARID2, ASXL1, ATM, ATRX, BCOR, BRAF, BRCA2, CCND2, CDK4/6, CDKN2A/B, CDKN2C, EGFR, EP300, FGFR1, KMT2A, KMTD2, MDM2/4, MET, MSH6, NF1, NOTCH1, PDGFRA, PIK3CA/3R1, PTEN, PTPN11, RB1, SETD2, SMARCA4, STAG2, TP53,* and *TSC2*. Arm level alterations were selected if they were significant in survival analyses as noted above or were canonically associated with adult-type gliomas: 1p loss, 4q loss, 7q gain, 10q loss, 11p gain, 12p gain, 14q loss, 15q loss, 19q loss, and 22q loss. Chromosome 9p loss was excluded from clustering analyses due to its collinearity with the loss of *CDKN2A/B*. UMAP clustering was performed on both non-TCGA and TCGA datasets, as the input was reported molecular profiles.

## Results

**Figure 1.**
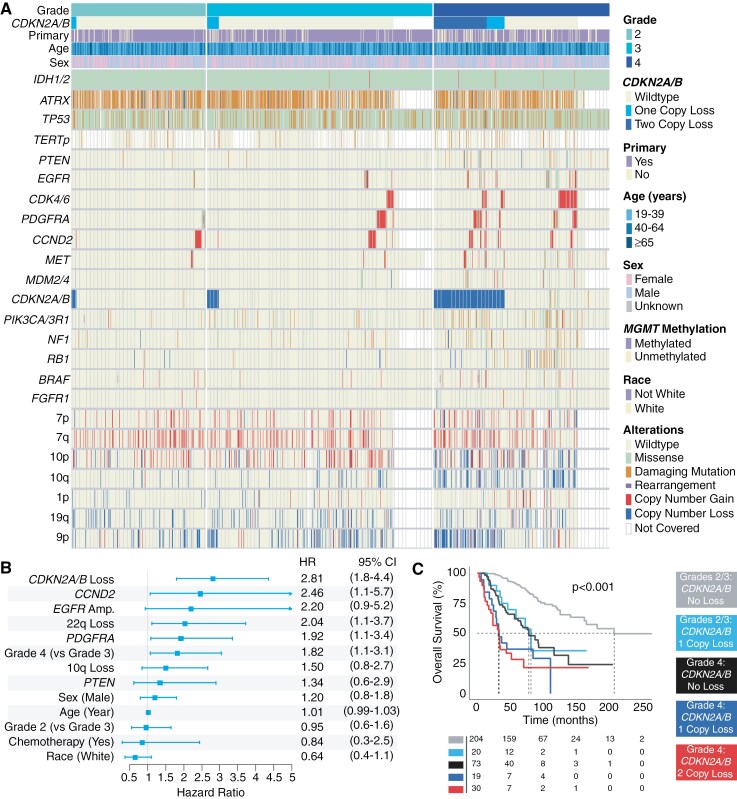
(A) Genomic signatures in *IDH1/2*-mutant astrocytomas, stratified by tumor grade. (B) Multivariate adjusted prognostic features in *IDH1/2*-mutant astrocytomas of all grades both negatively and positively influencing overall survival. (C) Overall survival of *IDH1/2*-mutant astrocytomas stratified by grade and *CDKN2A/B* status. Kaplan–Meier survival curves demonstrate that *CDKN2A/B* hemizygous loss is associated with worse survival in patients with *IDH1/2*-mutant astrocytomas of any grade.

We identified 998 unique adult patients with *IDH1/2*-mutant astrocytoma from three datasets: DFCI (*n* = 336), GENIE (*n* = 398), and TCGA (*n* = 264) (Figure 1A, Table 1, Supplementary Material 3). Patients had a median age of 37.3 years (mean ± SD: 39.0 ± 11.3 years, range: 19.2–78.1 years); 41.5% were female and 85.6% were white. There were 250 WHO grade 2 (25.1%), 420 grade 3 (42.1), and 328 grade 4 (32.9%) cases. 34 *IDH1/2*-mutant astrocytomas were upgraded to grade 4 based on the presence of *CDKN2A/B* homozygous-loss per WHO 2021 criteria (Table 1).^1^

**Table 1.** Patient Cohort

Variable	Grade 2	Grade 3	Grade 4	
Patients, *n*	250	420	328	
Cohort, *n* (%)				*P* < .001
TCGA	125 (50.0)	116 (27.6)	23 (7.0)	
DFCI/BWH	85 (34.0)	103 (24.5)	148 (45.1)	
Genie (v10)	40 (16.0)	201 (47.9)	157 (47.9)	
Sex (female), *n* (%)	114 (45.6)	175 (41.7)	125 (38.1)	*P* = .19
Primary tumor, *n* (%)	222 (88.8)	369 (87.9)	244 (74.6)	*P* < .001
Median age, years (range)	37.9 (19.2-77.2)	36.9 (20.0-75.0)	38.0 (20.0–78.1)	*P* = .11
Age (years), *n* (%)				*P* < .05
>19–39	140 (56.0)	248 (59.0)	175 (53.4)	
40–64	100 (40.0)	168 (40.0)	140 (42.7)	
≥65	10 (4.0)	4 (1.0)	13 (4.0)	
Race (white), *n* (%)	231 (94.7)	355 (91.0)	268 (87.3)	*P* = .12
Histopathologic grade, *n* (%)				*P* < .001
Grade 2	250 (100.0)	0 (0.0)	2 (0.6)	
Grade 3	0 (0.0)	420 (100.0)	28 (8.5)	
Grade 4	0 (0.0)	0 (0.0)	294 (89.6)	
High Grade	0 (0.0)	0 (0.0)	3 (0.9)	
Unknown	0 (0.0)	0 (0.0)	1 (0.3)	
Molecular alterations, *n* (%)				
* TERT* promoter	8 (5.5)	10 (4.1)	18 (10.5)	*P* = .03
* EGFR* amplification	0 (0)	4 (1.1)	12 (4.5)	*P* < .001
Whole Chr7 gain/Chr10 loss	0 (0)	2 (0.6)	6 (2.4)	*P* = .02
*CDKN2A/B* deletion				*P* < .001
Intact	241 (96.4)	326 (93.7)	137 (50.9)	
Hemizygous loss	9 (3.6)	22 (6.3)	33 (12.3)	
Homozygous loss	0 (0.0)	0 (0.0)	99 (36.8)	
*PDGFRA* amplification	1 (3.0)	17 (4.9)	26 (9.7)	*P* < .001
*CCND2* amplification	13 (5.2)	17 (4.9)	24 (8.9)	*P* = .09
*MDM2/4* amplification	0 (0)	0 (0)	6 (2.2)	*P* = .001
*MET* amplification	3 (1.2)	3 (0.9)	11 (4.1)	*P* = .01
*CDK4/6* amplification	0 (0)	13 (3.7)	46 (17.1)	*P* < .001
*ATRX*	176 (70.4)	257 (61.2)	186 (56.7)	*P* = .003
*TP53*	214 (85.6)	388 (92.4)	304 (92.7)	*P* = .005
*MGMT* methylated	37 (48.1)	51 (45.5)	67 (37.2)	*P* = .18

Summary of patient demographics, original histological diagnoses, and canonical molecular alterations associated with *IDH1/2*-mutant astrocytomas, stratified by grade. Percentages are calculated based on total number of samples assayed per variable. Chr: chromosome, n: number.

The overlap in clinical outcomes amongst grade 2 and 3 *IDH1/2*-mutant astrocytoma was readily evident, as grade 2 *IDH1/2*-mutant astrocytoma patients did not differ in overall survival from those with grade 3 tumors in the non-TCGA cohort (DFCI/GENIE; *P* = .88). Multivariate assessment of tumor grade further demonstrated this, with grade 2 tumor designation showing no significant impact on predicting survival when compared to grade 3 tumor designation (HR: 0.95, 95%CI: 0.6–1.6, Figure 1B). Grade 2/3 patients had median survival of 205.7 months while grade 4 median survival was 55.9 months.

### CDKN2A/B Loss and Focal Amplifications are Prognostically Significant

Amongst *IDH1/2*-mutant astrocytoma in the non-TCGA cohort, *CDKN2A/B* loss (hemizygous or homozygous) was the strongest negative prognostic feature (HR: 2.81, 95%CI: 1.8–4.4) after adjusting for demographic, molecular, and treatment variables (Figure 1B, Supplementary Material 3). The significance of *CDKN2A/B* loss on overall survival was consistently corroborated by internal validation (Supplementary Material 4) and within tumor grades (Supplementary Material 5).

Focal amplifications were also prognostically informative in *IDH1/2*-mutant astrocytomas with intact *CDKN2A/B* within the non-TCGA cohort (Figure 1B). *CCND2* and *PDGFRA* alterations, consisting mostly of focal amplifications, were disadvantageous independent of *CDKN2A/B* loss after multivariate adjustment (HR: 2.46, 95%CI: 1.1–5.7; HR: 1.92, 95%CI: 1.1–3.4 respectively).

### CDKN2A/B Hemizygous Loss Refines Histopathologic Grade

We next parsed the role of *CDKN2A/B* loss by looking at survival differences in cases with hemizygous loss. Amongst grade 2/3 *IDH1/2*-mutant astrocytomas in the non-TCGA cohort with available survival data, 8.9% of cases (20 of the 224) had a hemizygous loss of *CDKN2A/B* (Figure 1C) while 15.6% (19 of the 122) of grade 4 cases had a hemizygous loss of *CDKN2A/B*.

On univariate analysis, *IDH1/2*-mutant astrocytoma patients with *CDKN2A/B* hemizygous loss exhibited significantly shorter survival than their intact *CDKN2A/B* counterparts within each grade (Figure 1C). The median overall survival of grade 2/3 cases with *CDKN2A/B* hemizygous loss was 80.4 months, less than half of grade 2/3 cases with intact *CDKN2A/B* (median overall survival 205.7 months, *P* < .001). Grade 4 cases with either hemizygous or homozygous loss of *CDKN2A/B* also exhibited less than half the median overall survival (31.9 and 32.5 months, respectively) compared to grade 4 patients with intact *CDKN2A/B* (77.5 months, *P* = .09, *P* < .01 respectively). Of note, the median overall survival of grade 4 cases with intact *CDKN2A/B* was comparable with that of grade 2/3 cases with *CDKN2A/B* hemizygous loss (*P* = .75).

### Integrating CDKN2A/B and Focal Amplifications Define Prognostically Distinct Groups

**Figure 2.**
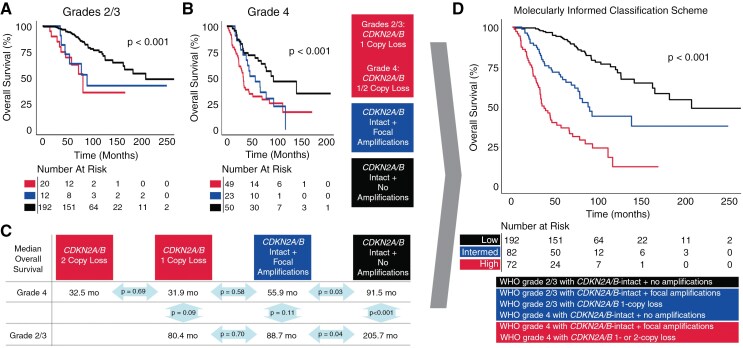
Kaplan–Meier survival curves of grade 2/3 (A) and grade 4 (B) *IDH1/2*-mutant astrocytomas demonstrate that canonical focal amplifications are associated with worse survival, independent of *CDKN2A/B* status. (C) Median overall survival of grade 2/3 and grade 4 *IDH1/2*-mutant astrocytomas, stratified by presence of *CDKN2A/B* loss or focal amplifications; *P*-values are of pairwise comparisons. (D) Kaplan–Meier survival curves of patients stratified across a molecular classification scheme incorporating amplifications, *CDKN2A/B* loss, and histopathologic grade, which partition patients into long-term, intermediate, and short-term survivors.

Given the prognostic role of both *CDKN2A/B* loss and focal amplifications, we sought to determine whether these two molecular markers could be integrated to refine the histopathologic stratification of *IDH1/2*-mutant astrocytoma. Within each grade, non-TCGA patients with intact *CDKN2A/B* and no focal amplifications survived the longest. Non-TCGA patients with grade 2/3 tumors that had intact *CDKN2A/B* and no focal amplifications exhibited a median overall survival of 205.7 months (Figure 2A). In comparison, median survival of grade 2/3 cases with *CDKN2A/B* hemizygous loss was similar to that of grade 2/3 cases with intact *CDKN2A/B* but focal amplifications (80.4 vs. 88.7 months, *P* = .70), and also similar to grade 4 cases with intact *CDKN2A/B* and no focal amplifications (91.5 months, Figure 2B-C, *P* = .93). Grade 4 *IDH1/2*-mutant astrocytomas with intact *CDKN2A/B* and focal amplifications showed decreased median survival at 55.9 months while grade 4 tumors with loss of *CDKN2A/B* (hemizygous: 31.9 months, homozygous: 32.5 months) fared the worst.

Compared to histopathologic grade alone, the combination of *CDKN2A/B* loss, focal amplifications, and histopathologic grade better defined a group of *IDH1/2*-mutant astrocytoma patients with intermediate overall survival (Figure 2D). In this molecularly informed classification scheme, low-risk or longer-term survivors were composed of grade 2/3 cases with intact *CDKN2A/B* and no focal amplifications (median overall survival: 205.7 months). Intermediate-risk or intermediate survival patients were those with (1) grade 2/3 tumors with intact *CDKN2A/B* and focal amplifications, (2) grade 2/3 tumors with *CDKN2A/B* hemizygous loss, or (3) grade 4 tumors with intact *CDKN2A/B* and no amplifications (median overall survival: 89.6 months). Finally, high-risk or shorter-term survivors possessed grade 4 tumors with focal amplifications and intact *CDKN2A/B*, *CDKN2A/B* hemizygous loss, or *CDKN2A/B* homozygous loss (median overall survival: 36.4 months, all comparisons *P* < .001).

### Molecular Subgroups Emerge With Distinct Outcomes

**Figure 3.**
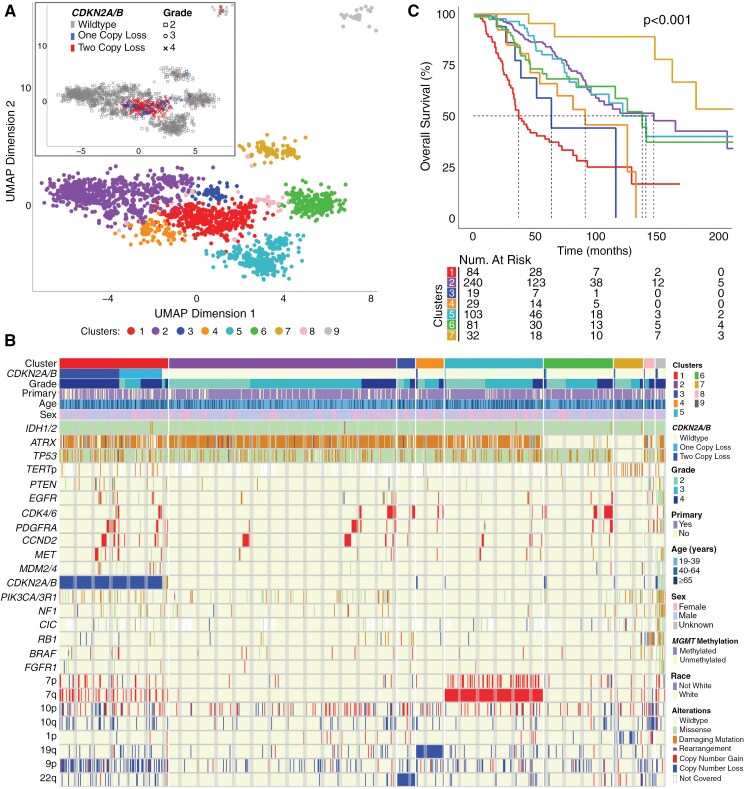
(A) Unsupervised clustering using UMAP and DBSCAN of *IDH1/2*-mutant astrocytomas revealed that samples partition primarily based on *CDKN2A/B*-hemizygous or homozygous loss. (B) Distinct genomic signatures of *IDH1/2*-mutant astrocytoma clusters. (C) Kaplan–Meier curves demonstrate that loss of *CDKN2A/B* is associated with the lowest median overall survival compared to other molecular subgroups of *IDH1/2*-mutant astrocytomas (*P* < .001). Kaplan–Meier curves could not be generated for clusters 8–9 due to limited sample size.

Unbiased molecular clustering from TCGA and non-TCGA cohorts revealed 9 distinct profiles across *IDH1/2*-mutant astrocytoma (Figure 3A). Nearly all tumors with *CDKN2A/B* homozygous or heterozygous loss (91.7%) clustered together, irrespective of tumor grade (cluster 1, Figure 3A). Differences in *ATRX* mutation, *TP53* mutation, and chromosome 7q, 19q, and 22q copy number alteration drove segregation across clusters 2–7 (Figure 3B and Table 2). Notably, cluster 7 was characterized by intact *ATRX* and *TP53*, reminiscent of the molecular profile of *IDH1/2*-mutant oligodendrogliomas. Whereas clusters 1–8 captured *IDH1/2*-mutant astrocytomas of all grades, cluster 9 consisted of grade 4 *IDH1/2*-mutant astrocytomas with high mutational burden.

**Table 2.** Unbiased Clustering of TCGA and Non-TCGA Patients Revealed Subgroups of *IDH1/2*-Mutant Astrocytomas With Distinct Molecular Features and Median Overall Survival (OS)

Variable	Cluster 1	Cluster 2	Cluster 3	Cluster 4	Cluster 5	Cluster 6	Cluster 7	Cluster 8	Cluster 9
Patients, *n*	153	320	25	39	138	97	41	15	14
Median OS, range (months)	36.7 (1.7–167.6)	145.8 (0.1–250.3)	63.1 (2.4–115.1)	90.5 (5.5–131.5)	139.8 (0.1–239.9)	136.8 (0.1–249.9)	NA (1.3–262.2)	NA (2.6–101.2)	NA (2.0–173.3)
Primary tumor, *n* (%)	106 (69.7)	274 (85.6)	22 (88.0)	34 (87.2)	119 (86.2)	86 (88.7)	34 (82.9)	13 (86.7)	8 (57.1)
Median age, months (range)	39.8 (22.5–78.1)	35 (19.2–77.2)	39.1 (24.5–66.2)	38.4 (21.8–67.6)	40.0 (21.8–74.0)	35.0 (20.3–68.0)	40.9 (22.0–77.2)	43.8 (21.6–70.7)	48.8 (23.0–67.3)
Age (years), *n*, %									
>19–39	73 (47.7)	220 (68.8)	12 (48.0)	22 (56.4)	65 (47.1)	61 (62.9)	17 (41.5)	7 (46.7)	5 (35.7)
40–64	74 (48.4)	96 (30.0)	11 (44.0)	16 (41.0)	67 (48.6)	34 (35.1)	22 (53.7)	7 (46.7)	8 (57.1)
≥65	6 (3.9)	4 (1.2)	2 (8.0)	1 (2.6)	6 (4.3)	2 (2.1)	2 (4.9)	1 (6.7)	1 (7.1)
Grade, *n* (%)									
Grade 2	10 (6.5)	114 (35.6)	9 (36.0)	10 (25.6)	56 (40.6)	27 (27.8)	23 (56.1)	1 (6.7)	0 (0.0)
Grade 3	25 (16.3)	157 (49.1)	9 (36.0)	20 (51.3)	67 (48.6)	44 (45.4)	13 (31.7)	5 (33.3)	0 (0.0)
Grade 4	118 (77.1)	49 (15.3)	7 (28.0)	9 (23.1)	15 (10.9)	26 (26.8)	5 (12.2)	9 (60.0)	14 (100.0)
Molecular alterations, *n* (%)									
*CDKN2A/B* deletion									
Intact	9 (75.9)	320 (100.0)	25 (100)	37 (94.9)	137 (99.3)	93 (95.9)	39 (95.1)	14 (93.3)	11 (78.6)
Hemizygous	60 (39.2)	0 (0.0)	0 (0.0)	0 (0.0)	1 (0.7)	2 (2.1)	1 (2.4)	0 (0.0)	0 (0.0)
Homozygous	84 (54.9)	0 (0.0)	0 (0.0)	2 (5.1)	0 (0.0)	2 (2.1)	1 (2.4)	1 (6.7)	3 (21.4)
7q gain	43 (28.1)	1 (0.3)	0 (0.0)	0 (0.0)	138 (100.0)	1 (1.0)	3 (7.3)	1 (6.7)	4 (28.6)
19q loss	18 (11.8)	13 (4.1)	1 (4.0)	39 (100.0)	15 (10.9)	7 (7.2)	5 (12.2)	1 (6.7)	1 (7.1)
22q loss	21 (13.7)	7 (2.2)	25 (100.0)	3 (7.7)	11 (8.0)	4 (4.1)	2 (4.9)	4 (26.7)	1 (7.1)
*ATRX*	115 (75.2)	318 (99.4)	21 (84.0)	39 (100.0)	109 (79.0)	3 (3.1)	0 (0.0)	9 (60.0)	12 (85.7)
*TP53*	137 (89.5)	300 (93.8)	25 (100.0)	38 (97.4)	132 (95.7)	96 (99.0)	1 (2.4)	14 (93.3)	12 (85.7)
*RB1*	8 (5.2)	9 (2.8)	3 (12.0)	1 (2.6)	1 (0.7)	0 (0.0)	2 (4.9)	11 (73.3)	9 (64.3)
10q loss	29 (19.0)	23 (7.2)	2 (8.0)	4 (10.3)	7 (5.1)	7 (7.2)	4 (9.8)	10 (66.7)	3 (21.4)
*CCND2* amp	18 (11.8)	23 (7.2)	1 (4.0)	0 (0.0)	8 (5.8)	2 (2.1)	0 (0.0)	0 (0.0)	0 (0.0)
*CDK4/6* amp	7 (4.6)	17 (5.3)	5 (20.0)	4 (10.3)	2 (1.4)	20 (20.6)	0 (0.0)	0 (0.0)	2 (14.3)
*EGFR* amp	6 (3.9)	3 (0.9)	0 (0.0)	1 (2.6)	1 (0.7)	3 (3.1)	0 (0.0)	2 (13.3)	0 (0.0)
*MDM2/4* amp	4 (2.6)	0 (0.0)	0 (0.0)	0 (0.0)	0 (0.0)	1 (1.0)	1 (2.4)	0 (0.0)	0 (0.0)
*MET* amp	9 (5.9)	4 (1.2)	0 (0.0)	1 (2.6)	3 (2.2)	0 (0.0)	0 (0.0)	0 (0.0)	0 (0.0)
*PDGFRA* amp	15 (9.8)	15 (4.7)	1 (4.0)	1 (2.6)	1 (0.7)	6 (6.2)	0 (0.0)	2 (13.3)	2 (14.3)

In addition to molecular segregation, tumor clusters varied in overall survival across both the TCGA and non-TCGA cohorts (Figure 3C). Cluster 1, defined by *CDKN2A/B* loss, demonstrated the shortest median overall survival (36.7 months), whereas cluster 7 which had oligodendroglial-like features (*ATRX* and *TP53* wild type) did not reach median survival by 250 months. Focal amplifications in particular did not drive cluster segregation but tended to enrich amongst higher grade *IDH1/2*-mutant astrocytoma cases within each cluster.

## Discussion

The integration and routine utilization of molecular classification for glioma has reshaped how we understand tumor behavior, counsel patients about prognosis, and assess clinical trials in neuro-oncology. Amongst glioma subtypes, *IDH1/2*-mutant astrocytoma has experienced one of the largest shifts in the transition from histopathologic to molecular classification, with almost half of these tumors previously classified as glioblastoma or other non-astrocytic gliomas.^3,7^ As a result, *IDH1/2*-mutant astrocytoma reflects a clinically heterogenous group, with a broad range of survival outcomes, especially amongst grade 2 and grade 3 tumors. We refine the molecular criteria for *IDH1/2*-mutant astrocytoma by augmenting histologic grade with hemizygous *CDKN2A/B* deletion and focal gene amplifications to establish three prognostically distinct tiers.^4,11,12^

The mechanisms by which hemizygous *CDKN2A/B* loss and focal amplifications impact tumor behavior continue to be investigated across gliomas and other cancer subtypes. *CDKN2A/B* encodes for tumor suppressors, preventing uncontrolled transitions through the cell cycle. Interestingly, in non-central nervous system cancers such as leukemia, it has been hypothesized that *CDKN2A/B* is haplo-insufficient, where hemizygous loss would not produce adequate production of protein necessary for tumor suppression.^21,22^ We see a similar phenomenon amongst *IDH1/2*-mutant astrocytoma, with one copy loss of *CDKN2A/B* demonstrating a significantly worse prognosis, which was similar to that of grade 4 tumors with two copy loss. There has been additional characterization of hemizygous *CDKN2A/B* loss in meningioma, with multiple studies demonstrating even one copy loss rapidly accelerates the time to meningioma recurrence.^23–25^ Collectively, these data support the expansion of molecular grading criteria for aggressive *IDH1/2*-mutant astrocytomas to include both hemizygous and homozygous *CDKN2A/B* loss.

Similarly, focal amplifications have strong biological underpinnings as a negative prognostic feature, with both intrachromosomal or extrachromosomal amplifications driving tumor growth.^26^ For example, *EGFR* amplifications have long been associated with aggressive cancer phenotypes and now are one of the defining alterations in *IDH*-wild-type glioblastoma.^1^ While some of these focal amplification events have been selectively assessed in *IDH1/2*-mutant astrocytoma previously, we see how many have a similar independent effect on survival amongst *IDH1/2*-mutant astrocytoma cases.^27,28^ For example, from our cohort, *CCND2* alterations (a majority of which were amplifications) and *EGFR* amplifications demonstrated a similar hazard ratio for overall survival (2.46 and 2.20, respectively). As such, the aggregation of multiple focal amplification events into a binary variable (amplified vs. not amplified) can enable a streamlined assessment of risk.

Integrating molecular markers that are readily accessible through routine profiling alongside histopathologic grade to better risk stratify patients has a direct translational impact on patients with *IDH1/2*-mutant astrocytoma. Recent clinical trial data from IDH inhibitors have sparked a new treatment paradigm for *IDH1/2*-mutant glioma patients, with the IDH inhibitors vorasidenib and ivosidenib demonstrating increased progression-free survival and delaying time to the next intervention.^29,30^ Trials and drug approval parameters have focused on histologic grade 2 *IDH1/2*-mutant gliomas without additional molecular characterization beyond 1p/19q status. Analysis of trial results alongside the classification criteria presented here (*CDKN2A/B* loss and focal amplifications) might further stratify which patients better respond to treatment and whether more aggressive *IDH1/2*-mutant astrocytomas could benefit from IDH inhibitors.^31^ In our analysis, 9 of the 250 grade 2 *IDH1/2*-mutant astrocytoma patients had hemizygous loss of *CDKN2A/B* and 13 of the 250 had an amplification in *CCND2*. Our findings suggest these patients may have distinct outcomes compared to their intact grade 2 counterparts. Similarly, we see the heterogeneity amongst grade 3 *IDH1/2*-mutant astrocytoma patients, many of whom have no *CDKN2A/B* alteration or focal amplification events and therefore may have a disease course that appears more “lower-grade.” This finding may be important in the context of IDH inhibitors given the observation that a subset of patients with grade 3 *IDH1/*2-mutant gliomas seemed to benefit from therapy.^6^ As such, classification schemes which more accurately define patient risk remain a critical component in the ongoing effort to develop targeted therapeutics that improve on current standards of care.

More broadly, the unique molecular classes which emerged across *IDH1/2*-mutant astrocytoma with distinct survival outcomes reinforce the biological variability amongst these gliomas and the need to consider individual patient profiles. The unbiased clustering methods we applied to *IDH1/2*-mutant astrocytoma did not include any demographic, histological, or clinical information. Nevertheless, we see how molecular phenotypes can segregate in overall survival, with certain tumor clusters, such as those without *ATRX* alteration, having a median survival of over three times that of tumors with *CDKN2A/B* alteration. We further observed a cluster of *IDH1/*2-mutant astrocytoma with oligodendroglioma-like features (group 7). These gliomas had rare *ATRX/TP53* alterations, frequent *TERT*-promoter/*CIC* alterations, and frequent chromosome 1p loss without co-occurring 19q loss. Notably, this group exhibited survival outcomes that appeared distinct from other *IDH1/*2-mutant astrocytoma clusters, not reaching median overall survival after 20 years of assessment, despite 44% of gliomas in this cluster being histopathologically classified as grade 3 or 4. Amongst other tumor clusters, we continued to see a distribution of histopathologic grade. For example, in the cluster of *IDH1/2*-mutant astrocytoma without *ATRX* alteration (cluster 6), while median overall survival was nearly 12 years, more than a quarter of tumors were grade 4. As we better understand the granularity of *IDH1/2*-mutant astrocytoma molecular alterations from population-level analyses, it becomes critical to consider the composition of each tumor’s unique molecular signature when counseling and managing these patients.

While we took steps to standardize and integrate the data across multiple streams, this study does have several limitations, some of which are tied to variability in data processing from public repositories. The large cohort of institutions included resulted in heterogeneous panels of genes assayed, with a more limited set of shared molecular features for analysis. Though WHO criteria guide the assessment of histopathologic grade, differences in pathologist review at the time of surgery and/or diagnosis across institutions can introduce additional variability. As data was collected over several decades, variability in data entry and reconciliation may impact the analyses in this study, though we reviewed all institutional data to verify clinical and molecular information. This includes limitations in reporting on last patient follow-up and overall survival. Finally, the focal amplifications included as part of this analysis represent a subset of prognostically significant amplifications described for *IDH1/2*-mutant astrocytomas, such as *MYCN*. Larger cohorts capturing rare amplifications as well as testing of amplification thresholds can help refine additional focal amplification events that can help stratify overall patient survival.

Assessment of molecular alterations such as *CDKN2A/B* hemizygous loss may also be dependent on the assays used and tumor purity of samples, though we approached these analyses as representative of real-world sequencing methodologies implemented in clinical practice. This was notable as the GENIE dataset had no cases noted with *CDKN2A/B* hemizygous loss, with cases potentially being considered *CDKN2A/B* intact. As more granular molecular markers approach clinical translation, it will be critical to examine the reliability of institutional methodologies for calling homozygous versus hemizygous loss. This includes validating sequencing platforms for hemizygous loss, performing sensitivity analyses to determine thresholds for calling hemizygous loss in bulk-sequenced samples, and differentiating between subpopulations of hemizygous and homozygous loss within the same tumor sample. Ensuring these criteria are reported amongst published and publicly accessible molecular data can help ensure the generalizability of such molecular markers.

Taken together, our findings support the existence of a clinically distinct group of *IDH1/2*-mutant astrocytoma with an intermediate prognosis, which can guide patient management and trial consideration. We believe the survival estimates and molecular characterization provided here will continue to guide management across this complex and heterogeneous group of patients.

## Supplementary material

Supplementary material is available online at *Neuro-Oncology* (https://academic.oup.com/neuro-oncology).

noae258_suppl_Supplementary_Material

## Data Availability

Data used in this study is available online through National Cancer Institute (NCI) Genomic Data Commons and Synapse. De-identified data for DFCI/BWH available as part of GENIE on Synapse.
