# Supplementary material for: Canonical amplifications and CDKN2A/B loss refine IDH1/2-mutant astrocytoma prognosis
Source: Neuro Oncol. 2024 Nov 25;27(4):993–1003. doi: 10.1093/neuonc/noae258 (PMC12083226; doi:10.1093/neuonc/noae258)
Supplement: noae258_suppl_Supplementary_Material [file noae258_suppl_supplementary_material.docx]

Table of Contents

[Supplement 1 – Institutional Datasets and Data Remediation 2](#_Toc175646474)

[Supplement 2 – Patient Survival in TCGA and Non-TCGA Cohorts 3](#_Toc175646475)

[Supplement 3 – Prevalence of Clinical and Molecular Features across Non-TCGA cohorts 4](#_Toc175646476)

[Supplement 4 – Internal Validation of “All Grades” Multivariate Adjusted Prognostic Features 5](#_Toc175646477)

[Supplement 5 – Prognostic Features of Grade 2/3 and Grade 4 IDH1/2-mutant Astrocytomas 6](#_Toc175646478)

# **Supplement 1 – Institutional Datasets and Data Remediation**

**TCGA:**

**Clinical and molecular data for 266 *IDH1/2*-mutant astrocytomas (matched tumor-normal samples) were downloaded from the National Cancer Institute (NCI) Genomic Data Commons (TCGA-Low Grade Glioma and TCGA-Glioblastoma datasets,** https://gdc.cancer.gov**). 264/266 cases met the age and grade criteria for inclusion (patient was a young adult (>19 years of age) and tumor was assigned grade 2, 3, or 4). Mutations, copy number variants (CNV), and structural variants (SV) were annotated for all *IDH1/2*-mutant astrocytoma cases. Across both datasets, 846 genes were assayed through whole exome sequencing. Chromosomal arm-level changes were identified through the Genomic Identification of Significant Targets in Cancer (GISTIC) module.**

**DFCI/BWH:**

**Molecular and Clinical data for 358 *IDH1/2*-mutant astrocytomas were retrieved from the institutional data retrieval system. Additional clinical data and MGMT methylation status were annotated through retrospective review of electronic medical records. 336/358 cases met age and grade criteria for inclusion. Patient follow-up was collected through July 1, 2023. Targeted next-generation sequencing (OncoPanel) was performed at the Center for Advanced Molecular Diagnostics at Brigham and Women’s Hospital. OncoPanel results reported mutational status, CNVs, and SVs. Number of genes assayed increased with each updated Oncopanel version (277 genes (OncoPanel V1), 302 genes (OncoPanel V2), or 477 genes (OncoPanel V3).**

**We developed a proprietary pipeline to remove germline variants and isolate somatic alterations in these unmatched tumor samples. First, we removed variants with either 1) allele frequency >0.1% in the Genome Aggregation Database (https://gnomad.broadinstitute.org) or 2) variants annotated as benign in the NIH ClinVar database (https://ncbi.nlm.nih.gov/clinvar). Second, the excluded variants were re-added to the somatic dataset if they were present in the Catalogue of Somatic Mutations in Cancer (COSMIC, https://cancer.sanger.ac.uk/cosmic). Chromosomal arm-level changes were identified using the Arm-level Copy-number Events in Targeted Sequencing (ASCETS, Spurr et al., 2020:** <https://doi.org/10.1093/bioinformatics/btaa980>**) platform developed at DFCI. Chromosome arms were considered amplified or deleted if >70% of the chromosome arm was altered.**

GENIE:

Clinical and molecular data for **574** ***IDH1/2*-mutant astrocytomas** were downloaded from Synapse; the GENIE repository was filtered for *IDH1/2*-mutant astrocytoma cases (https://synapse.org/genie). Samples from DFCI/BWH were filtered out. 398/574 cases met age and grade criteria for inclusion. Across the 15 institutions that contributed *IDH1/2*-mutant astrocytoma data, there were 23 distinct targeted-sequencing panels. 8 of these gene panels provided CNVs and 4 provided SVs. Gliomas without sufficient molecular data to be classified as *IDH1/2*-mutant astrocytoma were excluded (1p19q status cannot be ascertained and ATRX/TP53 intact or not available). The contributing institutions developed internal pipelines to remove germline variants; additionally, the American Association of Cancer also applied an internal germline filtering pipeline. We generate chromosomal arm level calls through ASCETS; we used the same arm call threshold as for DFCI/BWH (>70% chromosome arm altered). MGMT methylation data and survival data was only available for cases contributed by Memorial Sloan Kettering Cancer Center (GENIE-MSK). GENIE v13 (April 2023) provided overall patient survival and follow up durations.

Glioma Classification and Data Processing:

If 1p/19q status could not be ascertained, the presence of *ATRX* or *TP53* mutations indicated it was likely an astrocytoma. We removed duplicated samples and samples with incomplete genomic profiles. Among patients with multiple samples, we selected the earliest occurring tumor. Genes included in downstream analyses were assayed in at least one panel per cohort.

# **Supplement 2 – Patient Survival in TCGA and Non-TCGA Cohorts**


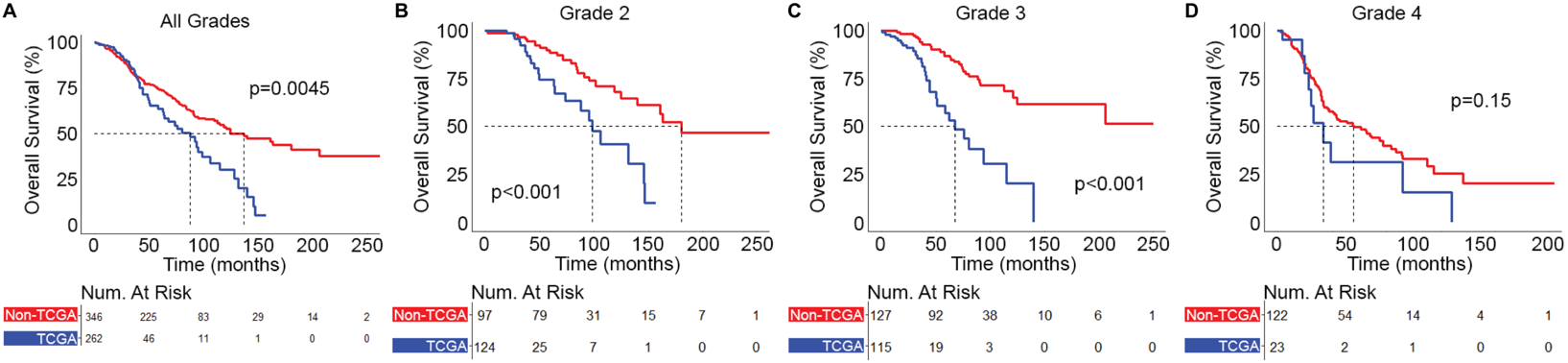


**Supplement 2:** Kaplan-Meier curves demonstrate that non-TCGA patients have significantly longer overall survival than TCGA patients when considering (A) all, (B) grade 2, and (C) grade 3 *IDH1/2*-mutant astrocytomas (p<0.005). (D) Median overall survival for patients with grade 4 *IDH1/2*-mutant astrocytomas in contemporary institutional cohorts was longer compared to than those in the TCGA cohort but this did not reach statistical significance.

# **Supplement 3 – Prevalence of Clinical and Molecular Features across Non-TCGA cohorts**

**Supplement 3:** Demographic, clinical, and molecular summary of non-TCGA patients, with pairwise calculations between DFCI and GENIE datasets. Percentages are calculated based on total number of samples assayed per variable; n: number.

| Variable | DFCI | | GENIE | p-value |
| --- | --- | --- | --- | --- |
| Patients, n | 336 | | 398 |  |
| Grade, n (%) |  | |  | p<0.001 |
| Grade 2 | 85 (25.3) | | 40 (10.1) |  |
| Grade 3 | 103 (30.7) | | 201 (50.5) |  |
| Grade 4 | 148 (44.0) | | 157 (39.4) |  |
| Sex (Female), n (%) | 152 (45.2) | | 144 (36.2) | p=0.02 |
| Median Age, years (range) | 38.3 (20.5-78.1) | | 37.0 (20.0-75.0) | p=0.32 |
| Primary Tumor, n (%) | 239 (71.3) | | 332 (83.4) | p<0.001 |
| Age (years), n (%) |  | |  | p=0.28 |
| >19-39 | 179 (53.3) | | 232 (58.3) |  |
| 40-64 | 146 (43.5) | | 158 (39.7) |  |
| ≥65 | 11 (3.3) | | 8 (2.0) |  |
| Race (White), n (%) | 301 (90.4) | | 306 (88.2) | p=0.42 |
| Histopathologic Grade, n (%) | |  |  | p<0.001 |
| Grade 2 | 87 (25.9) | | 40 (10.1) |  |
| Grade 3 | 110 (32.7) | | 222 (55.8) |  |
| Grade 4 | 138 (41.1) | | 133 (33.4) |  |
| High Grade | 1 (0.3) | | 2 (0.5) |  |
| Unknown Grade | 0 (0.0) | | 1 (0.3) |  |
| Molecular Alterations, n (%) |  | |  |  |
| *CDKN2A/B* deletion |  | |  | p<0.001 |
| Intact | 235 (69.9) | | 220 (82.4) |  |
| Hemizygous loss | 58 (17.3) | | 0 (0.0) |  |
| Homozygous loss | 43 (12.8) | | 47 (17.6) |  |
| *CCND2* amplification | 10 (3.0) | | 16 (6.0) | p=0.11 |
| *CDK4/6* amplification | 29 (8.6) | | 21 (7.9) | p=0.85 |
| *EGFR* amplification | 7 (2.1) | | 6 (2.2) | p=1.00 |
| *MDM2/4* amplification | 4 (1.2) | | 1 (0.4) | p=0.52 |
| *MET* amplification | 5 (1.5) | | 3 (1.1) | p=0.98 |
| *PDGFRA* amplification | 11 (3.3) | | 20 (7.5) | p=0.03 |

# **Supplement 4 – Internal Validation of “All Grades” Multivariate Adjusted Prognostic Features**


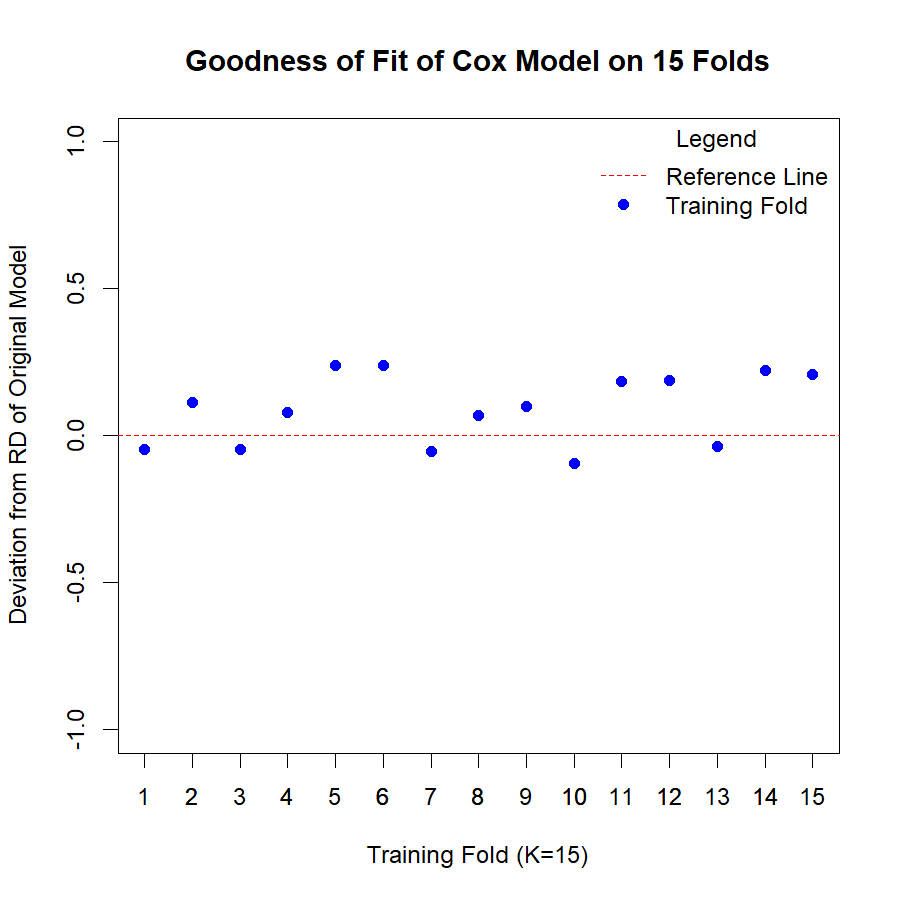


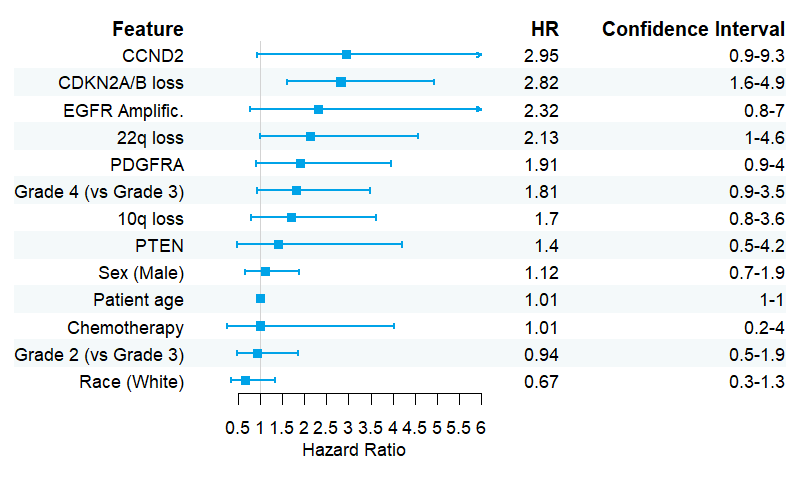


Mean Hazard Ratio of 15 Test Sets of “All Grades“

**Supplement 4:** Mean Hazard Ratios (HR) and mean 95% confidence intervals (CI) of multivariate adjusted prognostic features from Figure 1B. The multivariate model for all grades (Figure 1B) was validated on 15 randomized subsets of non-TCGA patients; each subset was n= 225 (65% of original dataset, n = 346) without replacement. Pseudo-R-squared (RD) deviation of each testing folds compared to the multivariate model run on the non-TCGA dataset in Figure 1B.

# **Supplement 5 – Prognostic Features of Grade 2/3 and Grade 4 *IDH1/2*-mutant Astrocytomas**


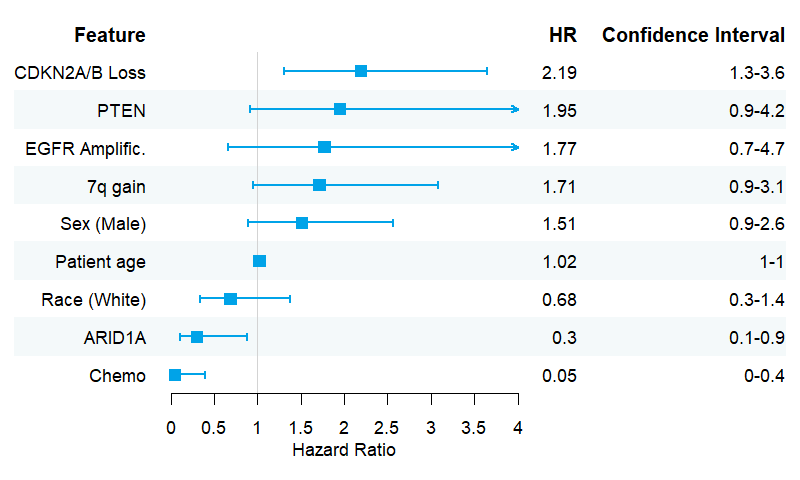


Grade 4 *IDH1/2*-mutant Astrocytomas


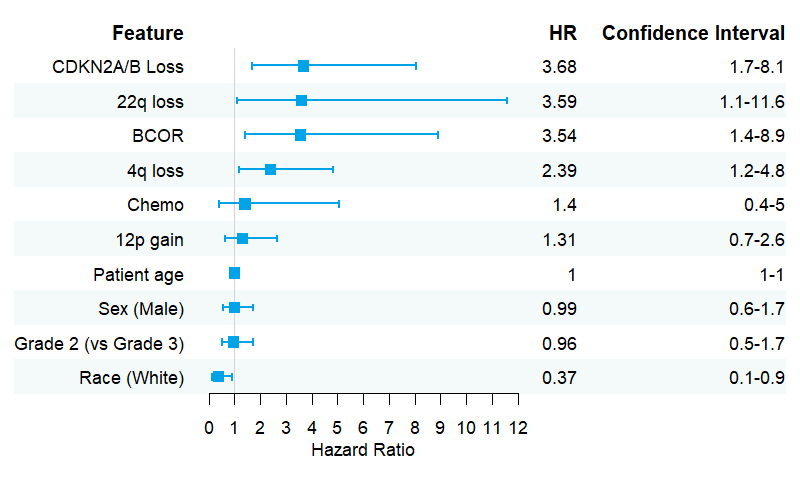


Grade 2/3 *IDH1/2*-mutant Astrocytomas

**Supplement 5:** Hazard ratios (HR) and 95% confidence intervals (CI) of multivariate adjusted prognostic features across non-TCGA *IDH1/2*-mutant astrocytomas that are (A) grade 2/3 and (B) grade 4.
